# Increased levels of Stress-inducible phosphoprotein-1 accelerates amyloid-β deposition in a mouse model of Alzheimer’s disease

**DOI:** 10.1186/s40478-020-01013-5

**Published:** 2020-08-21

**Authors:** Rachel E. Lackie, Jose Marques-Lopes, Valeriy G. Ostapchenko, Sarah Good, Wing-Yiu Choy, Patricija van Oosten-Hawle, Stephen H. Pasternak, Vania F. Prado, Marco A. M. Prado

**Affiliations:** 1 Robarts Research Institute, The University of Western Ontario, 1151 Richmond St. N., London, Ontario N6A 5B7 Canada; 2 Program in Neuroscience, The University of Western Ontario, 1151 Richmond St, London, N6A 3K7 Canada; 3 School of Molecular and Cell Biology and Astbury Centre for Structural Molecular Biology, University of Leeds, Leeds, LS2 9JT UK; 4 Department of Biochemistry, Schulich School of Medicine & Dentistry, The University of Western Ontario, Medical Sciences Building, 1151 Richmond St. N, London, N6A 5B7 Canada; 5 St. Joseph’s Health Care London-Parkwood Institute, St. Joseph’s Hospital, 268 Grosvenor St Room A1-015, London, N6A 4V2 Canada; 6 Department of Clinical Neurological Sciences, Schulich School of Medicine & Dentistry, 1151 Richmond St, London, N6A 3K7 Canada; 7 Department of Anatomy & Cell Biology, The University of Western Ontario, 1151 Richmond St, London, N6A 3K7 Canada; 8 Department of Physiology and Pharmacology, Schulich School of Medicine and Dentistry, The University of Western Ontario, 1151 Richmond St, London, N6A 3K7 Ontario Canada

**Keywords:** Amyloidosis, Alzheimer’s disease, STIP1/HOP, Hsp70, Hsp90, Prion protein

## Abstract

Molecular chaperones and co-chaperones, which are part of the protein quality control machinery, have been shown to regulate distinct aspects of Alzheimer’s Disease (AD) pathology in multiple ways. Notably, the co-chaperone STI1, which presents increased levels in AD, can protect mammalian neurons from amyloid-β toxicity in vitro and reduced STI1 levels worsen Aβ toxicity in *C. elegans*. However, whether increased STI1 levels can protect neurons in vivo remains unknown. We determined that overexpression of STI1 and/or Hsp90 protected *C. elegans* expressing Aβ_(3–42)_ against Aβ-mediated paralysis. Mammalian neurons were also protected by elevated levels of endogenous STI1 in vitro, and this effect was mainly due to extracellular STI1. Surprisingly, in the 5xFAD mouse model of AD, by overexpressing STI1, we find increased amyloid burden, which amplifies neurotoxicity and worsens spatial memory deficits in these mutants. Increased levels of STI1 disturbed the expression of Aβ-regulating enzymes (BACE1 and MMP-2), suggesting potential mechanisms by which amyloid burden is increased in mice. Notably, we observed that STI1 accumulates in dense-core AD plaques in both 5xFAD mice and human brain tissue. Our findings suggest that elevated levels of STI1 contribute to Aβ accumulation, and that STI1 is deposited in AD plaques in mice and humans. We conclude that despite the protective effects of STI1 in *C. elegans* and in mammalian cultured neurons, in vivo, the predominant effect of elevated STI1 is deleterious in AD.

## Introduction

Alzheimer’s disease (AD), the most common cause of dementia, afflicts close to 50 million people worldwide and the number of affected individuals is projected to grow by twofold every two decades [1]. Genetic mutations that cause AD favour the abnormal processing of the amyloid precursor protein (APP), from which the aggregation prone amyloid-β (Aβ) peptide is derived [2–5]. In sporadic and familial AD, Aβ peptides accumulate in soluble and diffuse oligomeric forms and are deposited in insoluble extracellular plaques. Over the past two decades it is becoming increasingly apparent that soluble oligomeric forms of Aβ are a major toxin driving other pathologies and inflammation in AD [6–14], supporting the “amyloid hypothesis” [15, 16]. These small Aβ peptides attack neurons causing neuronal dysfunction many years prior to the appearance of cognitive symptoms, by interacting with distinct neuronal proteins and receptors.

One of the key receptors involved in Aβ toxicity is the prion protein (PrP^C^), which interacts with Aβ oligomers (AβOs) with high-affinity, and this interaction triggers metabotropic glutamate receptor 5 (mGluR5) maladaptive signalling in neurons [17–20]. PrP^C^ functions as an extracellular scaffolding protein that interacts with multiple ligands and receptors [21–23]. Specifically, Stress-inducible phosphoprotein 1 (STI1, STIP1, or mammalian homolog: Hsp organizing protein, HOP), a Hsp70/Hsp90 co-chaperone, once secreted binds to PrP^C^, and protects mouse neurons against a number of insults [24–28]. In vitro, extracellular STI1 prevents toxic effects of AβOs, including neuronal death and decreased long-term potentiation, likely by interfering with AβO-PrP^C^ interaction due to contiguous binding sites for STI1 and AβOs along PrP^C^ [29]. Conversely, functional studies using a *C. elegans* model of Aβ indicate that knockdown of Hsp90, STI1 and several other co-chaperones increases Aβ toxicity [30]. Notably, STI1 has been identified in a genome-wide transcriptome analysis as one of the top genes regulating ER transcriptome stress response in the brains of sporadic AD patients [31], and STI1 protein levels are upregulated in AD patient brains [29].

In addition to interacting with PrP^C^, STI1 has a conserved role as a co-chaperone for Hsp90 and Hsp70, and disruption of STI1 function significantly interferes with Hsp70/Hsp90 client expression and activity [25, 32–34]. STI1 overexpression rescued mutant huntingtin proteotoxicity in yeast by reorganizing cytotoxic huntingtin into large high-molecular weight foci [35], suggesting that increased STI1 levels could regulate amyloid aggregation and lessen amyloid toxicity in mice by multiple mechanisms. Molecular chaperones such as Hsp70 and Hsp90 also play critical roles in AD and other protein misfolding diseases [36–39]. In neurodegenerative diseases, there is considerable evidence that molecular chaperone complexes are altered [40] and Hsp90 inhibition in vivo reduced neuronal and synaptic loss due to amyloid toxicity in mice [41]. Moreover, blocking abnormal chaperome interactions have also been shown to improve tau toxicity [40].

Given that STI1 seems to prevent multiple proteotoxic effects in yeast [35, 42, 43]), *C. elegans* [30, 44] and mammalian neurons [26, 28, 29, 45], we hypothesized that increased STI1 levels can mitigate Aβ toxicity and aggregation in vivo. We tested this assumption by modulating levels of STI1 in a *C. elegans* model of Aβ toxicity, in cultured mouse neurons, and by overexpressing STI1 (3-fold) in the 5xFAD mouse model of AD. In *C. elegans*, STI1 and Hsp90 protected worms against Aβ-induced paralysis. We also determined protection of cultured mammalian neurons against AβOs by endogenous extracellular STI1. In contrast, raising STI1 levels in mice amplified the formation of extracellular Aβ plaques, which increased neurodegeneration. Surprisingly, we found STI1 can accumulate in extracellular plaques in mice and it was also found in mature plaques in humans. Our results suggest a complex relationship between STI1 and amyloid toxicity and indicate that some of the protective effects observed in *C. elegans* may not directly translate to mammalian systems in vivo, due to the more complex mechanisms of amyloidosis in the latter.

## Materials and methods

### Ethics statement

Animals were housed at The University of Western Ontario vivarium and were managed and treated according to the Canadian Council of Animal Care (CCAC) guidelines and Animal Use Protocols (2016–103, 2016–104). Human tissue from autopsy of AD patients was obtained with informed consent and approval from Office of Research Ethics, Protocol 162656E at the University of Western Ontario. Patients were assigned a neuropathological diagnosis by a licenced Neuropathologist certified by the Royal College of Physicians and Surgeons of Canada.

### Nematode strains and growth conditions

*C. elegans* were grown on Nematode Growth Medium (NGM) plates seeded with the *E. coli* OP50–1 strain and cultured by standard methods [46]. The *C. elegans* strain expressing Aβ_(3–42)_ in the body wall muscle (CL2006 (*dvIs2 [pcL12(unc-54/human Aβ peptide minigene) + pRF4*]) was obtained from the *Caenorhabditis* Genetics Center. Strains overexpressing HSP-90 in the body wall muscle *(AM988 (rmIs347(unc-54p::HSP-90::RFP]* [47] and the strain overexpressing STI-1 in the muscle (PPI1972 (*unc-54p::STI-1::GFP*); a kind gift of Dr. Anat Ben-Zvi, Ben Gurion University, Israel) were crossed into the genetic background of CL2006, resulting in strains PVH50 *(*AM988 *(rmIs347(unc-54p::HSP-90::RFP]; dvIs2)*, and PVH40 (PPI1972 (*unc-54p::STI-1::GFP);dvIs2)*, respectively. Overexpression of HSP-90 and STI-1 together in the Aβ_(3–42)_ expressing strain was achieved by crossing strain AM988 into the genetic background of PVH40, resulting in strain PVH71 (*rmIs347(unc-54p::HSP-90::RFP);*(*unc-54p::STI-1::GFP);dvIs2).*

### Paralysis assays

An age-synchronised population of 100 animals per strain was scored by monitoring movement via the touch-nose response, using a platinum wire. Animals were transferred to fresh OP50–1 plates or indicated RNAi plates every day. For paralysis assays on RNAi plates, age-synchronised *C. elegans* were transferred onto *hsp-90* RNAi plates at L4 stage to avoid any developmental effects, and onto *sti-1* RNAi plates at L1 stage.

### Primary hippocampal neuronal cultures

Neuronal cultures from E17.5 WT control and TgA pups were generated as described previously [29, 48]. Briefly, hippocampi were separated from dissected brains clear of meninges, and the cells were dissociated using 0.25% trypsin-EDTA solution (ThermoFisher, Cat#25200056). Approximately 4–6 × 10^4^ cells were added to each well in 4-well plates (ThermoFisher, Cat#176740) pre-coated with Poly-L-Lysine (Catalog# P6407, Sigma). Cells were plated in Plating medium containing 10% heat inactivated fetal bovine serum (Catalog# 12484–028, Gibco), 4.5% glucose (Catalog# A2494001, Invitrogen), 1% Sodium Pyruvate (Catalog# 11360070, Invitrogen), 1% Pen/Strep (Catalog# 11360070, Invitrogen) and 1% GlutaMAX (Catalog# 35050–161, Invitrogen) in MEM with Earle’s BSS (Catalog # 11095, Invitrogen). After 4 h at 37 °C and 5% CO_2_, plating media was removed and replaced with complete Maintenance Medium (Neurobasal Medium, 1% Pen/Strep, 1% GlutaMAX and 1% B27 supplement (Catalog# 17504044, Gibco). Half of media were replaced with fresh medium every 2 days and experiments were performed after 10–12 days in culture.

### Cellular viability assays

AβO toxicity was assessed in hippocampal neuronal cultures as described previously [17, 29, 48]. Briefly, neurons were plated in 4-well dishes coated with Poly-L-Lysine at a seeding density of 1 × 10^5^. AβOs were added to 10–12 day old cultures to the final concentration of 1 μM. After that cultures were incubated for 24–48 h at 37 C, 5% CO_2_, and the neuronal health was assessed by various methods.

Lactate Dehydrogenase release was assessed in control and TgA hippocampal neurons as described previously [29] using Lactate Dehydrogenase (LDH) Activity Assay Kit (Catalog # MAK066, Sigma). Briefly, neuronal cultures were grown in a phenol red-free medium for 10 days, treated with AβOs for 24 h, after which the culture media were collected and cleared by centrifuging for 20 min at 1000 x g and 4 °C. LDH activity was quantified by measuring absorbance at 450 nm using BioRad iMark Plate Reader.

LIVE/DEAD Viability/Cytotoxicity assay was performed according to the manufacturer's instructions (Cat#L3224, ThermoFisher). Briefly, 10–11 day old neuronal cultures were treated with AβOs with or without anti-STI1 antibody (1:500, Bethyl Laboratories, [25]) for 48 h, after which the cells were washed with PBS and incubated for 30 min in the dark with Calcein-AM and Ethidium homodimer-1. Cultures were then immediately imaged using 488 nm laser for excitation and 505/20 emmission filter for Calcein and 560/50 emmission filter for Ethidium homodimer-1 using an LSM 510 Confocor 2 confocal microscope equipped with 10x /0.3 objective.

### Mouse line generation and husbandry

STI1 overexpressing mice, TgA, were generated on a C57BL6/J background as described previously [25, 49]. The 5xFAD Alzheimer’s disease mouse line (B6.Cg Tg [APPSwFlLon,PSEN1*M146L*L286V] 6799Vas/J RRID:MGI:034840-JAX) was obtained from The Jackson Laboratory. Mice were crossed to generate TgA-5xFAD and 5xFAD littermate progeny and maintained on a mixed background of B6SJLF1/J and C57BL6/J, with no further backcrossing. Male mice were used for behavioural and immunohistochemical experiments because previous characterization of TgA mice that was necessary for this work was mainly done in male mice. Mice used in experiments were given ad libitum access to food (Harlan) and water. Mice were housed in standard plexiglass cages with 2–4 littermates. Room temperature and humidity were controlled at 22–25 °C and with 40–60% respectively, and the light schedule followed a light/dark cycle from 7 am-7 pm.

### Preparation and purification of Aβ peptides

Aβ_1–42_ (rPeptide, Catalog# 1002) were prepared as monomer films as described previously [29] and stored desiccated at − 80 °C until use, for up to 2 months. Oligomers were generated and characterized from Aβ_1–42_ films as described previously [29]. Briefly, peptide films were thoroughly dissolved in DMSO to make a 1 mM stock, after which the solution was diluted 10-fold in Ham’s F12 Medium (Wisent), centrifuged for 10 min at 16,100 x g and then incubated for 24 h at 4 °C. Preparation quality was regularly checked by SDS-PAGE, probing with the anti-Aβ (6E10, BioLegend) antibody. Oligomers and control preparations were either used directly or frozen at − 80 °C in aliquots and used for up to 4 weeks.

### Morris water maze

The spatial Morris water maze (MWM) task was performed as described previously [32, 49–51]. Seven to eight WT (TgA^-^-5xFAD^-^, one mouse excluded for reversal phase of task), twelve 5xFAD and twelve TgA-5xFAD male mice were used for behavioural analyses. No a priori power estimates were used, but numbers were chosen based on previous experiments following the MWM protocol [32, 49–51]. We used 6-month-old mice because in previous experiments 5xFAD male mice at this age have been shown to cross the platform less, take longer to find the platform and have spatial reference memory deficits compared to WT C57BL6 controls [52].

Experiments were performed at 22–24 °C in a room with two large lamps next to the pool illuminating the room, and animals were acclimated for approximately 30 min before task initiation. Performance on the MWM was recorded with ANY-Maze Software. The researcher was blind to genotypes during experiments, however, analyses for reference memory, reversal memory and acquisition of the task were not done blindly. Animals were trained for four days, four trials per day, to find the platform in the water tank. On the fifth day of testing (probe trial), the platform was removed and the percentage of time during 60 s that a mouse spent in each quadrant was analyzed, and this is referred to as reference memory/probe trial. Two days following the acquisition and memory testing phase, animals started the reversal phase of the task, in which the platform was moved to a different quadrant. Learning to acquire the new position of the platform was assessed again over four days, and reversal memory after the platform was removed on the fifth day was analyzed as described above.

### Western blotting

After mice (3–5-month-old, male) were anesthetized with ketamine (100 mg/kg)-xylazine (20 mg/kg), the animals were perfused with ice cold PBS and one whole hemisphere was collected for neuropathology (procedure described below) and the other hemisphere was dissected for Western Blotting or ELISA. Tissue was dissected on ice to isolate cortex and hippocampus, then flash frozen on dry ice before transferred to − 80 °C for long-term storage. Western blotting was performed as described previously [32, 49, 53]. Briefly, tissue was weighed and homogenized in ice cold RIPA buffer (50 mM Tris, 150 mM NaCl, 0.1% SDS, 0.5% Sodium Deoxycholate, 1% Triton-X 100, pH 8.0) with phosphatase inhibitors (1 mM NaF and 0.1 mM Na_3_VO_4_) and protease inhibitor cocktail (1:100, Catalog# 539134-1SET, Calbiochem), followed by cold sonication 3 × 7 s. Homogenates were then rocked at 4 °C for 20 min, then centrifuged at 10,000 x g for 20 min at 4 °C to isolate protein. Protein was quantified using BioRad DC Protein assay (Catalog# 5000112, BioRad). 5–80 μg of protein was loaded onto 4–12% Bis-Tris Plus Gels (ThermoFisher) or on 13.5% Tris-Tricine Gels. Protein was transferred onto PVDF membranes (Catalog #: IVPH00010, EMD Millipore) by BioRad Semi-Dry Transfer Trans-Blot Turbo system. Primary antibodies used for immunoblotting: anti-STI1 (1:5000, in-house antibody generated by Bethyl Laboratories Montgomery, USA), anti-Hsp90β (1:1000, Cat#5087, Cell Signaling, RRID:AB_10548761), anti-Hsp70 (1:1000, Cat#ab2787, Abcam, RRID:AB_303300), anti-APP (C-terminal; 1:1000, Cat#ab32136, Abcam, RRID:AB_2289606), anti-APP (N-terminal; 1:1000, Cat#ab2072, Abcam, RRID:AB_302812), anti-BACE1 (1:1000, Cat#5606, Cell Signaling, AB_1903900), anti-MMP2 (1:1000, Cat#ab37150, Abcam, RRID:AB_881512), Anti-CD10 (Neprilysin: 1:1000, Cat#ab951, Abcam, RRID:AB_2146533), Anti-Insulin Degrading Enzyme (1:1000, Cat#ab32216, Abcam, RRID:AB_775686), anti-PSD95 (1:1000, Cat#MA1046, Pierce, RRID:AB_2092361), anti-Synaptophysin (1:1000, Cat#5461, Cell Signaling, RRID:AB_10698743). Loading control used was anti-β-actin (1:25000, Cat#A3854, Sigma, RRID:AB_262011) and secondary antibodies were sheep anti-mouse HRP (1:5000, Cat#SAB3701095, Sigma, RRID: N/A) and goat anti-rabbit HRP (1:10000, Cat#170–6515, BioRad, RRID:AB_11125142). Proteins were visualized using chemiluminescence on FluoroChemQ chemiluminescent exposure system (Alpha Innotech) or ChemiDoc MP Imaging System (BioRad) and analyzed using their respective software (Alpha Innotech and Image Lab).

### Biochemical fractionation to isolate Tris-soluble, membrane bound and insoluble APP

Tris-soluble, membrane-bound and insoluble fractions were isolated as described previously [50, 54]. Cortices from 3 to 5-month-old male mice perfused with ice cold phosphate-buffered saline (PBS), pH 7.4, were used for experiments. Tissue was weighed and homogenized using Wheaton Overhead Stirrer (Cat#903475) in 6.5× the weight of each sample in 20 mM Tris-HCl (pH 8.5) with protease and phosphatase inhibitors. Homogenates were centrifuged for 1 h at 135,000 x g at 4 °C. Supernatant was collected as Tris-soluble fraction and stored for ELISA at − 80 °C. Next, tissue was homogenized and resuspended in 10 mM Tris, 150 mM NaCl, 0.02% Triton-X 100 (pH 7.6) with protease and phosphatase inhibitors, 15× the weight of pellet, using Cordless Motor and Pestle (Cat#47747–370, VWR). Samples were centrifuged at 100,000 x g for 1 h at 4 °C. Supernatant was collected as the membrane-bound fraction and stored immediately at − 80 °C for Western blotting. Finally, pellet was resuspended and homogenized in 1 ml of 3 M Guanidinium Hydrochloride (GHCl), 50 mM Tris (pH 8.0) with protease inhibitors followed by vigorous vortexing and overnight incubation on a tube rotator at 4 °C to dissolve pellet into solution. This constituted the insoluble APP fraction, which was then stored at − 80 °C for use in ELISA.

### Human Aβ_1–42_ ELISA

ELISA for Tris-soluble and GHCl insoluble Aβ was performed as described previously [50, 54, 55] following manufacturer’s instructions (Catalog# KHB3441, ThermoFisher).

### Histological processing

After mice were anesthetized with ketamine (100 mg/kg)-xylazine (20 mg/kg) and then transcardially perfused with ice cold PBS (pH 7.4), one hemisphere was placed in 4% paraformaldehyde for 24 h, then in 15, 20% then 30% sucrose for 24 h at 4 °C before being embedded in OCT (Thermo Scientific Shandon Cryomatrix embedding resin, Catalog # 67–690-06), frozen on dry ice then stored at − 80 °C. Frozen tissue blocks were acclimated to − 20 °C for at least 30 mins prior to cryosectioning on Leica CM350 or CM1950 Cryostat, and 30 μM sections were collected and stored free floating in PBS + 0.02% sodium azide at 4 °C until use.

### Congo red

Free floating sagittal sections (30 μm) were mounted onto SuperFrost Plus Slides (Fisher), dried at RT, then stained following IHC World “Modified High pH Congo Red Staining Protocol for Amyloid” [50]. Briefly, sections were incubated in filtered 0.3% Congo Red Solution (dissolved in 80% ethanol with 1% sodium hydroxide) for 10 min in the dark. Sections were rinsed in distilled water until water ran clear, followed by 10 dips in alkaline alcohol (1% sodium hydroxide in 50% ethanol) solution, then water wash. Sections were counterstained in Harris Hematoxylin (Cat#10143–610, VWR) for 45 s, rinsed in water for 2 min and then rocked in tap water with 5 drops of concentrated (14.5 M) ammonium hydroxide until sections turned blue. After another water rinse, sections were dehydrated in 70, 95 and 100% ethanol, followed by two 100% xylene washes and slides were cover-slipped using DPX mounting media (Catalog# 44581, Sigma). Sections were imaged using Zeiss Axioskop Optical Microscope at 20X magnification, with two images captured sequentially (non-overlapping) along the dentate gyrus (from the apex to the hilus/opening of the blades), and one image each along the CA3, CA1 and subiculum. Sections were at least 120 μm apart, with 4–6 sections per animal being imaged and quantified. Experimenters were blind to genotype during image collection and analyses. Images were analyzed in Fiji (ImageJ, NIH), using the same parameters for each image and genotype. Images collected were converted to 8-bit, background was subtracted to create a light background and separate colours. Images were then deconvoluted using the H-PAS colour deconvolution function to analyze Congo Red staining more accurately. The percent area was averaged across the whole hippocampus (averaging the values obtained from each separate hippocampal sub-region) and then 4–6 sections per mouse were averaged, with 4–5 mice per genotype.

### Thioflavin S staining

Thioflavin staining was performed as described previously [54], following the AlzForum protocol (Alzforum https://www.alzforum.org/protocols/thioflavin-s-staining) with some modifications. Briefly, mounted 30 μm sagittal sections were incubated with 1% Thioflavin S (dissolved in 50% ethanol) for 8 min in the dark at room temperature. Upon removal of stain, sections were washed twice with 80% ethanol, 3 min each, then once with 95% ethanol for another 3 min. Sections were then re-hydrated with three distilled water washes. Sections were mounted using Immumount (Cat#9990412, ThermoScientific, Shandon) and imaged using LSM Meta 510 confocal microscope equipped with a 10X/0.3 Plan Neofluar objective. One-two sequential (non-overlapping) images along the dentate gyrus were captured, as well as 1 image each of the CA3, CA1 and posterior CA1/subiculum areas. For analyses, images were converted to 8-bit in Fiji, then a deconvoluted thresholded image was obtained with a white background and black plaques. The percent area of the plaques was measured using the Fiji Measure plugin, averaged across the whole hippocampus (averaging the values obtained from each separate hippocampal sub-region image), and then 4–6 sections per mouse of each genotype were averaged. Sections from 4 to 5 mice per genotype were used, and experimenters were blind to genotype during image collection and analyses.

### Silver staining

Silver staining was performed as described previously. Using 6-well plates and net-wells, free-floating sections were stained with the NeuroSilver™ staining kit II (Catalog#: PK301, FD NeuroTechnologies, Inc., Baltimore, USA) following manufacturer’s instructions. This kit labels degenerating neuronal bodies, processes and terminals. Images were taken using Zeiss Axioskop Optical Microscope at 20X magnification, with two images being taken along the dentate gyrus, from the apex to the hilus/opening of the blades, one image each of the CA3, CA1 and subiculum. At least 4 sections from 4 to 5 animals/genotype were stained and sections selected were at least 120 μm apart. Using ImageJ (Fiji) Software (NIH), images were converted to 8 bit and thresholded to make the silver particles black and background white (Circularity of 0–0.65). Particles were numbered and averaged for each animal. The same parameters were used for each section, animal and both genotypes.

### Immunofluorescence

Immunostaining for Aβ in mouse tissue was performed as described previously [54]. Sagittal 30 μm sections were mounted onto Superfrost Plus slides and after drying, sections were boiled in 10 mM sodium citrate (pH 6.1) for 20 min in antigen retriever at 95 °C, and then cooled on ice to RT for 1 h. Sections were then washed twice for 5 min in 1X Tris-buffered saline (TBS), pH 7.5. Next, sections were permeabilized by incubating in 0.3% Triton-X in TBS, pH 7.5, twice for 5 min and blocked in 2% horse serum (HS), 2% normal goat serum (NGS), 1% BSA, 0.3% Triton-X in TBS, pH 7.5, for 90 min at room temperature. Sections were then incubated with primary antibody anti-β-amyloid (6E10, Cat#803001, 1:200, Biolegend) alone, or with anti-STI1 (Bethyl Laboratories, 1:400), anti-Iba1 (Cat#:019–19,741, 1:1000, Wako, RRID:AB_839504) or anti-Hsp90β (Cat#5087, 1:50, Cell Signaling, RRID:AB_10548761). The following day after ~ 18 h incubation with primary antibody at 4 °C, sections were washed twice with TBS, 5 min each, then incubated with secondary donkey anti-rabbit Alexa Fluor 647 (1:500, Cat#A-31573, ThermoFisher) and/or goat anti-mouse Alexa Fluor 488 (1:500, Catalog# A-11001, ThermoFisher) antibodies for 2.5–3.5 h at 4 °C. Sections were washed three times with TBS then incubated with Hoechst 33342 (Cat#62249, ThermoFisher) for 15 min, rinsed three times, then mounted on slides using Immumount. For STI1 labelling with Aβ, prior to nuclear labelling, sections were incubated with Vector TrueVIEW™ Autofluorescence Quenching Kit (Cat#VECTSP8400, Vector) following manufacturer’s instructions. Immunofluorescent labelling for cleaved caspase-3 was performed as described previously [50]. Percent area analyses for Aβ immunoreactivity across the whole hippocampus were performed as described above for Thioflavin S staining. Images were captured using Leica TCS SP8 (Leica Microsystems Inc., Ontario, Canada) confocal system at 10X objective (N.A. 0.4) for Aβ percent area experiments, at 20X objective (N.A. of 0.75) for CC-3 immunostaining, and at 63X (N.A. of 1.4) for Aβ, STI1, Hsp90β and Iba1 imaging.

For analyses of Iba1+ microglia in contact with Aβ plaques, two sequential non-overlapping images along the dentate gyrus were captured, as well as 1 image of the CA3, 1 image of the CA1 and 1 image of the posterior CA1/subiculum. The number of microglia in each field of view was counted, considering a total (those surrounding/touching plaques and not associated (near/in contact) with plaques), and those only in contact with plaques. The number of Aβ plaques in each imaged subfield was counted. The sum number of plaque-associated microglia (those with processes or cell body seen touching plaque or no more than 10 μm from plaque) averaged per section and the average number of microglia around plaques were estimated. Images were captured using the using Leica TCS SP8 (Leica Microsystems Inc., Ontario, Canada) confocal system (40X objective, N.A. of 1.3).

### Human AD tissue and immunofluorescence

Blocks of parietal and temporal human brain tissue from 5 patients with a Neuropathological diagnosis of AD (all Braak stage 5–6: 3 male/2 female, average age 82; 75 year old (y/o) male, 88 y/o male, 89 y/o male, 80 y/o female, 82 y/o female), were embedded in paraffin and sections were cut at 5 μm using Microm HM335E Microtome, then deparaffinized using the Leica Autostainer XL. Sections were then left rocking in TBS, pH 7.6 for 2 h. Antigen retrieval was performed by boiling sections at 95 °C in 10 mM citrate buffer + 0.05% Tween (pH 6.0) for 20 min then cooled on ice in the same buffer for 1 h. Additionally, sections were incubated in 70% formic acid for 10 min then rinsed with 10% formic acid. Sections were rinsed with water, washed twice with TBS for 5 min. Next, tissue was permeabilized in TBS with 0.3% Triton X-100 for 5 min, followed by a 1.5 h blocking at RT in 2% horse serum, 2% NGS, 1% BSA, 0.3% Triton X-100 in TBS. Sections were incubated overnight at 4 °C with primary antibodies anti-STI1 (1:300, Bethyl) and anti-6E10 (1:100, Biolegend) in 1% horse serum, 1% NGS, 0.5% BSA and 0.1% Triton X-100 in TBS. The following day, sections were left at RT for ~ 2 h in primary antibody, then washed twice with TBS with 0.1% Triton X-100, followed by incubation with secondary antibodies donkey anti-rabbit Alexa Fluor 647 (Cat#A-31573, ThermoFisher, RRID:AB_2536183) and goat anti-mouse Alexa Fluor 488 (Cat#A-11001, ThermoFisher, RRID:AB_2534069). Sections were incubated with secondary antibodies for 4 h at 4 °C, then washed three times with TBS. After washing, the sections were then incubated for 30 s with TrueBlack Lipofuscin Autofluorescence Quencher according to manufacturer’s instructions (Cat#10119–144, Biotium), rinsed three times with TBS, and mounted on slides with Immumount. Images were obtained using Olympus FV1000 confocal microscope at 63X (N.A. of 1.35) at 1.5–2.5X zoom. Representative images shown are all from the same magnification and zoom.

### Statistical analysis

For *C. elegans* experiments, all experiments were repeated at least three times (3 biological replicates). Data are presented as mean values +/− SEM. *p*-values for two group comparisons were calculated using student’s t-test. (*) denotes *p* < 0.05; (**) denotes *p* < 0.01 and (***) denotes *p* < 0.0001. To compare two independent populations (paralysis assays) p-values were calculated using the Wilcoxon Mann-Whitney rank sum test. For mouse pathology and Western blot experiments, data were analyzed using student’s t-test, except for APP processing in which Two-Way ANOVA was used. For MWM Two-Way ANOVA with Repeated Measures were used to analyze datasets, with appropriate post-hoc analyses as required. Prism 7 software was used for all statistical analyses.

## Results

*STI1 and Hsp90 protect C. elegans and mouse primary hippocampal neurons against Amyloid-*β toxicity.

**Fig. 1:**
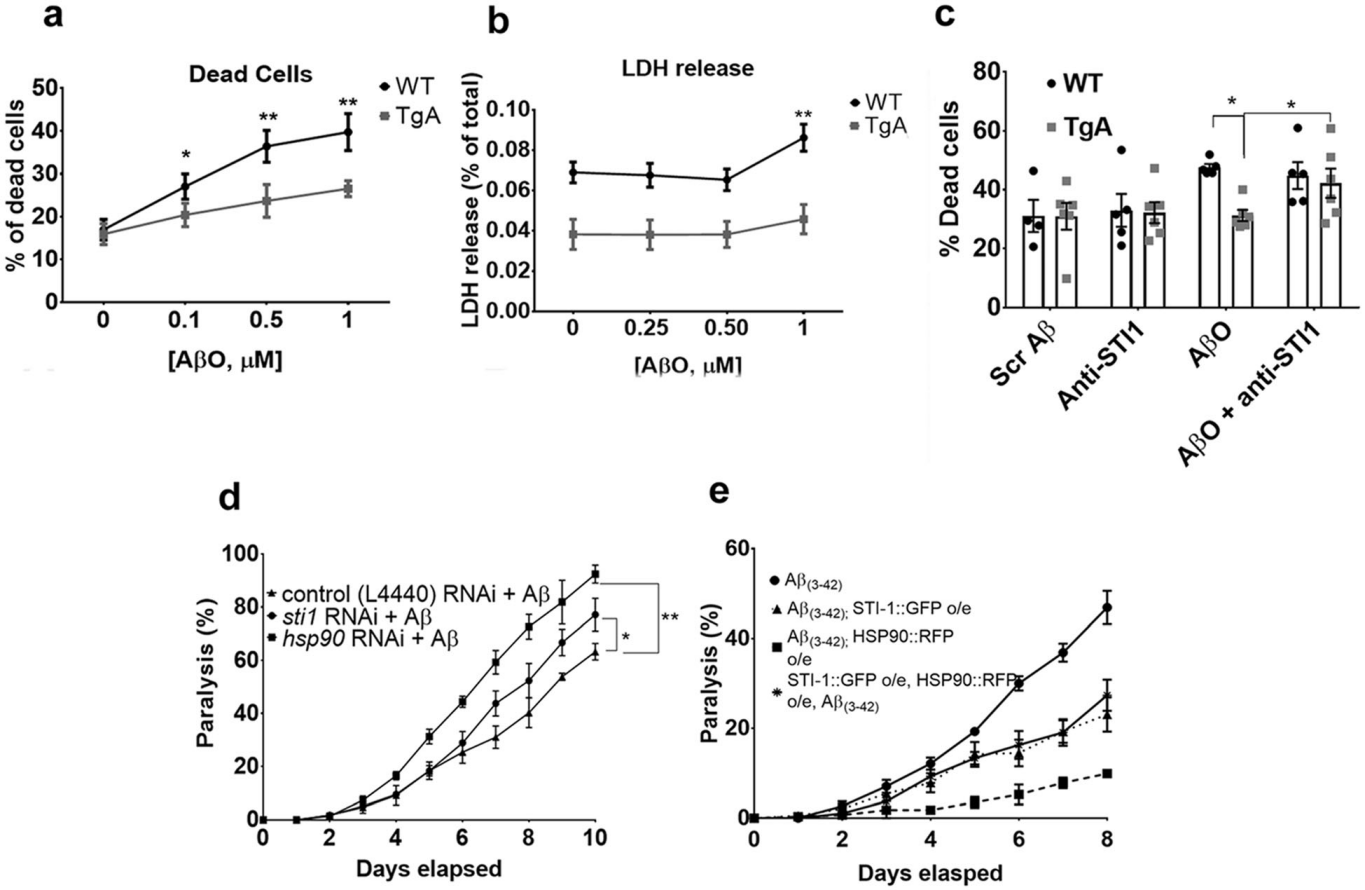
Overexpression of STI1 protects both *C. elegans* and primary hippocampal mouse neurons from Aβ-toxicity. **a** The average percent of dead cells ((# of dead cells)/(# of live + # of dead cells))× 100) in E17.5 primary hippocampal neuronal cultures from WT or TgA embryos. Cells were imaged in 4 well dishes from 8 random fields (*N* = 6 individual embryos/genotype). Dishes were treated with either no Aβ, 0.1, 0.5 or 1 μM AβOs for 48 h. **b** Likewise, WT or TgA primary hippocampal neurons were treated with 0, 0.25, 0.5 or 1 μM for 48 h, and LDH release was measured using colorimetric assay, read at 450 nm. *N* = 3–4 individual embryos for WT hippocampal cultures for each condition and *N* = 5 individual embryos per condition for TgA hippocampal cultures. **c** Quantification of percentage of cell death in hippocampal neuronal cultures treated for 48 h with 1 μM scrambled Aβ control, antibody against STI1 (1:500), AβO alone (1 μM) or dishes treated with both AβO (1 μM) and anti-STI1 (1:500). At least four individual embryos were used for each condition and genotype. **d** Percentage of body paralysis over 10 days in nematodes expressing Aβ_(3–42)_ (strain CL2006 (dvIs2 [pcL12*(unc-54/human Aβ peptide minigene) + pRF4*]) in the bodywall muscle and treated with empty vector control RNAi (black triangle), *sti-1* RNAi (black circle), or *hsp-90* RNAi (black square). **e** Percentage of paralysis in worms expressing Aβ_(__3–42)_ (black circle), Aβ_(__3–42)_ worms overexpressing HSP90 in body wall (strain AM988 (rmIs347(*unc-54p::HSP-90::RFP*)) (black square), Aβ_(__3–42)_ worms overexpressing STI-1 in muscle cells (strain PVH40 (PPI1972 (*unc-54p::STI-1::GFP);dvIs2*))) (black triangle) and Aβ_(3–42)_ worms overexpressing both STI-1 and HSP-90 in the bodywall muscle (strain PVH71 (rmIs347(*unc-54p::HSP-90::RFP*);(*unc-54p::STI-1::GFP);dvIs2*) (black stars). For *C. elegans* experiments, 100 age synchronized animals were used for analyses. For panels a and b, data were analyzed using Two-Way ANOVA, with Sidak’s or Bonferroni’s post-hoc tests for multiple comparisons, respectively, comparing WT vs TgA across the different concentrations of the AβOs. For both panels c and d, groups were analyzed using Wilcoxon statistics, comparing to Aβ_(3–42)_ expressing organisms. *p < 0.05, ** *p* < 0.01. All data are mean ± SEM. Raw data available for Figure 1: 10.6084/m9.figshare.12115614

We have previously shown that treatment with recombinant STI1 can protect cultured neurons from AβO induced cell death, by preventing AβO mal-adaptative signalling via the prion protein [29]. To investigate whether increased endogenous expression of STI1 can protect mammalian neurons from AβO toxicity, we derived primary hippocampal neuronal cultures from E17.5 mouse wild-type (WT) control embryos and those endogenously overexpressing STI1 (mouse line named TgA; overexpressing STI1 3-fold [25]). The cultures were subjected to increasing concentrations of AβOs for 48 h. At 0.5 μM and 1.0 μM of AβOs, neuronal cultures from TgA mice died significantly less than control cultures (Fig. 1a, *p* = 0.025, *p* = 0.019 for 0.5 μM and 1.0 μM, respectively), determined using the Live/Dead Assay. Likewise, using the LDH assay, TgA neuronal cultures released less LDH (indicator of dying cells) than control neurons at 1.0 μM of AβOs (Fig. 1b, *p* = 0.0017). Since exogenous recombinant STI1 and PrP^C^ interaction blocks AβO toxicity [29], we tested whether secreted STI1 in TgA primary neurons was responsible for increased neuronal resilience against AβOs. We blocked extracellular STI1 signalling with a validated STI1 antibody [25] and assessed neuronal survival in the presence of AβOs using the Live/Dead Viability and Toxicity kit. We found that treating control or TgA cultures with the anti-STI1 antibody had no significant effect on cell viability (Fig. 1c, *p* > 0.1), in cultures pre-treated with 1 μM AβOs, TgA neuronal cultures had significantly less cell death than control cultures (Fig. 1c, *p* = 0.033). However, WT and TgA neurons treated with AβOs and the anti-STI1 antibody displayed elevated levels of cell death, and TgA neurons were no longer resilient against AβO insult (Fig. 1c, p > 0.1).

In addition to preventing toxic AβO signalling [29], STI1 can modulate proteostasis [32, 42]. In yeast, Hsp70 requires STI1 for the reorganization of amyloid-like proteins into toxicity buffering foci [35], and Hsp70 and Hsp90, which are bridged by STI1, can decrease Aβ fibril formation and aggregation [56]. Hence, to initially study STI1 and Hsp90 mediation of Aβ proteotoxicity, we used *C. elegans*, an organism that does not express the prion protein or its homologues, but has been shown to require STI1 for protection and reduction of amyloid aggregates [30].

Previous work has shown that knockdown of STI-1 and HSP-90 in *C. elegans* increases Aβ toxicity [30]. We repeated and expanded these experiments, and confirmed in *C. elegans* expressing Aβ_(3–42)_ that RNAi-mediated knockdown of *sti-1* or *hsp-90* significantly reduced motility compared to worms only expressing Aβ_(3–42)_ over 10 days (Fig. 1d, *p* = 0.0195, *p* = 0.0078, for *sti-1* and *hsp-90*, respectively). Next, we tested whether overexpression of STI-1 and HSP-90 in worms could reduce Aβ-induced paralysis. We found that worms overexpressing HSP-90 seven-fold (*C. elegans homolog: DAF-21/HSP-90*,Aβ_(3–42)_; HSP-90::RFP) were less paralysed than Aβ_(3–42)_ worms (Fig. 1e, *p* = 0.016). Furthermore, worms overexpressing STI-1 two-fold (Aβ_(3–42)_; STI-1::GFP) displayed decreased levels of paralysis (46% vs 23% at day 8, p = 0.016, Fig. 1e). Interestingly, overexpression of both HSP-90 (7-fold) and STI-1 (2 fold; Aβ_(3–42)_; STI-1::GFP; HSP-90::RFP) alleviated paralysis to a similar degree as overexpression of STI-1 alone (46% vs 27% at day 8, p = 0.016), and showed a lesser effect than overexpression of HSP-90 by itself. Hence, in a genetic worm model of Aβ toxicity, both STI-1 and HSP-90 have protective effects, although their effects were not synergistic.

### Elevated amyloid deposition in 5xFAD mice overexpressing STI1

**Fig. 2:**
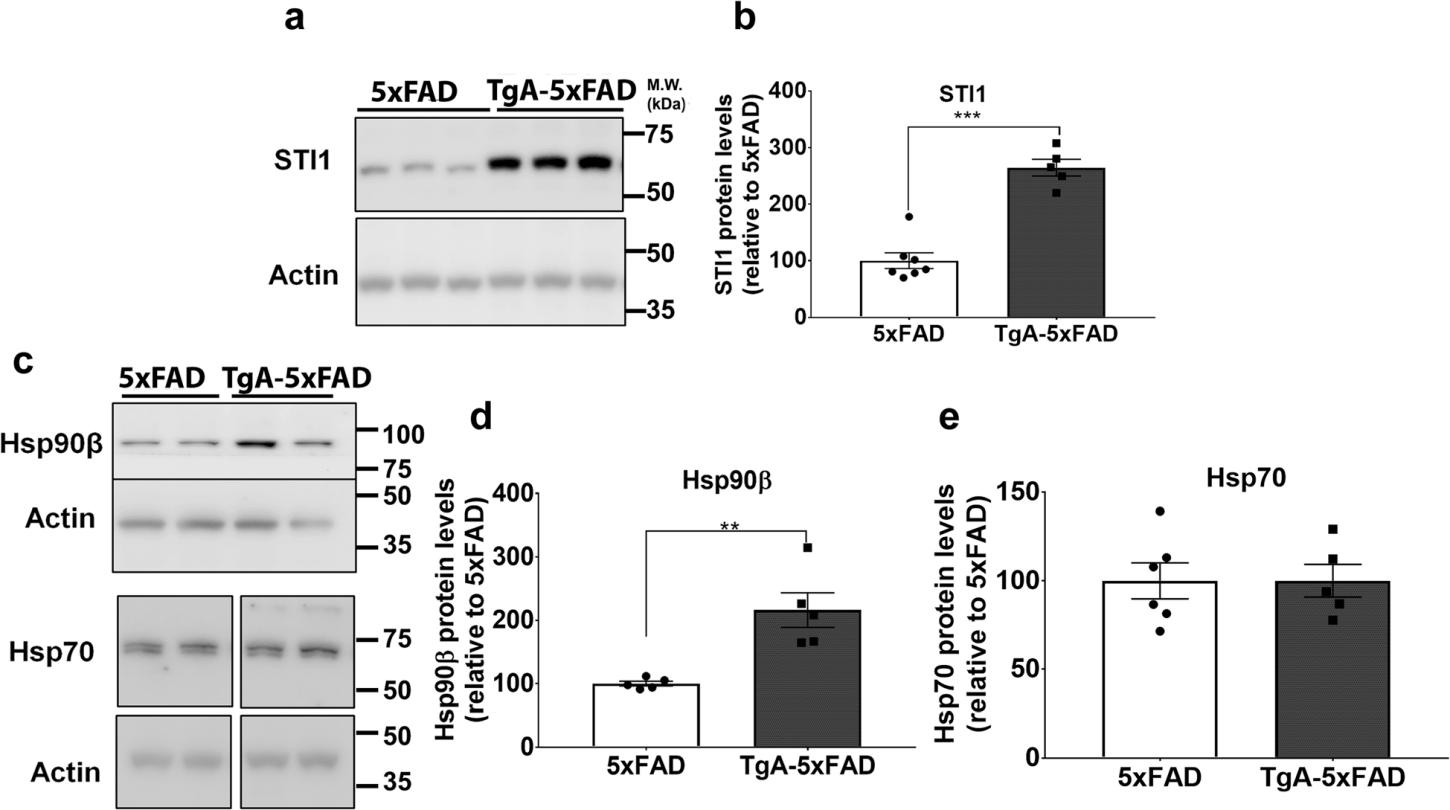
Elevated levels of STI1 and Hsp90β in STI1 overexpressing 5xFAD mice (TgA-5xFAD). **a** Representative immunoblots from hippocampal lysates obtained from 3 to 5-month-old male 5xFAD and TgA-5xFAD mice for STI1. **b** Protein levels of STI1 relative to actin and normalized to 5xFAD control. **c** Representative immunoblots and quantification from hippocampal lysates obtained from 3 to 5-month-old male 5xFAD and TgA-5xFAD mice for **d** Hsp90β (previously shown to be increased in plain TgA mice) **e** and Hsp70. All data are mean ± SEM and were analyzed using unpaired t-test. At least 5 mice were used for each genotype. Experiments were repeated at least twice. *p < 0.05, **p < 0.01, ***p < 0.0001. Raw data available at: 10.6084/m9.figshare.12115704

To test whether the observations from cultured neurons and *C. elegans* occur in vivo in an AD model, we crossed the 5xFAD mouse line with the STI1 overexpressing mice, TgA. We compared the changes in TgA-5xFAD mice with their littermate controls 5xFAD for all subsequent experiments. We first confirmed that STI1 was indeed overexpressed 3-fold as previously reported in the original TgA line (Fig. 2). Similar to what we observed in the original TgA mice (C57BL/6 background) [49], the constitutive Hsp90 isoform, Hsp90β, was increased in the TgA-5xFAD mice in comparison to 5xFAD littermate controls (Fig. 2a, b, *p* < 0.0001, Fig. 2c, d, *p* = 0.027). The elevated levels of Hsp90 are likely a compensatory response to increased STI1 levels [49], supporting a strong genetic relationship between Hsp90 and STI1 [57]. Moreover, we found no difference in Hsp70 protein levels between 5xFAD and TgA-5xFAD mice (Fig. 2c, e, *p* > 0.1).

**Fig. 3:**
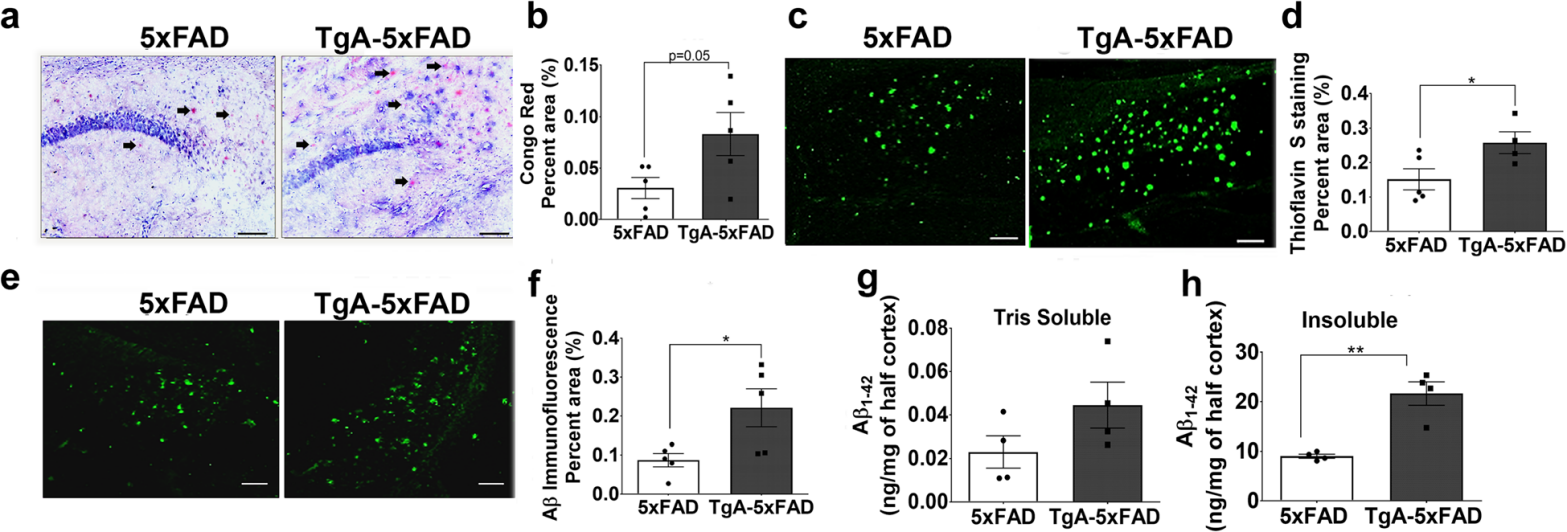
Increased amyloidosis in young male 5xFAD mice overexpressing Hsp90 co-chaperone STI1. **a** Representative brightfield micrographs of Congo Red labelling in the CA1/Subiculum, at 10X magnification and **b** quantification of average area fraction (% area) of Congo Red staining across the whole hippocampus in 3–5-month-old male 5xFAD and TgA-5xFAD mice. Black arrows indicate plaques labelled with Congo Red. On each graph, black circles represent individual 5xFAD mice and black squares are individual TgA-5xFAD mice. **c** Thioflavin-S staining representative images from CA1/Subiculum hippocampal subfield and **d**, quantification of average percent area of the whole hippocampus with Thioflavin S staining. **e** Aβ (6E10 antibody, Biolegend) immunoreactivity in CA1/Subiculum and **f** quantification of average percent area across the whole hippocampus with Aβ immunofluorescence. Concentration (ng/mg) of Tris Soluble **g**, and GHCl Insoluble **h**, Human Aβ_1–42_ in cortex (1 hemisphere) from 3 to 5-month old 5xFAD and TgA-5xFAD male mice. All scale bars = 25 μm. Data are presented as mean ± SEM, and experiments were analyzed using unpaired t-test. *p < 0.05, ** p < 0.01. Raw data available at: 10.6084/m9.figshare.12115713

We then tested whether STI1 overexpression would affect Aβ deposition in 5xFAD mice. Since Hsp90 and Hsp70 reduce Aβ fibrillization [56] and concerted Hsp70-STI1 reorganizes diffuse amyloid [35], we predicted that overexpressing STI1 would reduce Aβ aggregation and toxicity. We first evaluated amyloidosis using Congo Red (labelling β-pleated sheets in amyloid fibrils [58]), and Thioflavin S (labelling β-pleated sheets in amyloid fibrils and extracellular neuritic plaques, [59]). Surprisingly, we detected two-fold increase in Congo red amyloid labelling in the hippocampi of 3–5-month-old TgA-5xFAD male mice compared to 5xFAD mice (Fig. 3a, b, *p* = 0.05). Likewise, when we examined Thioflavin S staining, there was significantly more amyloid deposition in the hippocampi of TgA-5xFAD mice (Fig. 3c, d, *p* = 0.032). Moreover, Aβ immunoreactivity in TgA-5xFAD mice, detected using an anti-Aβ antibody (6E10, which also labels APP, intracellular and extracellular amyloid), was double that of 5xFAD mice (Fig. 3e, f, *p* = 0.047). We further evaluated the degree of Aβ accumulation in 5xFAD and TgA-5xFAD cortices using ELISA against Human Aβ_1–42_. We performed these analyses in the cortex, since both the cortex and hippocampus show considerable Aβ accumulation by 3–5 months of age in 5xFAD mice [60]. In congruence with the Aβ stains and immunolabelling in hippocampi, we detected an increase in GHCl insoluble Aβ, but not Tris-Soluble Aβ_1–42_ levels (*p* > 0.14 for soluble and *p* = 0.0020 for insoluble, Fig. 3g, h) in TgA-5xFAD male mice compared to 5xFAD controls. Surprisingly, these experiments suggest that STI1 overexpression combined with the increase in Hsp90 levels were linked to early accumulation of Aβ insoluble plaques and augmented levels of insoluble amyloid.

### Reduced neuronal resilience in 5xFAD mice overexpressing STI1

**Fig. 4:**
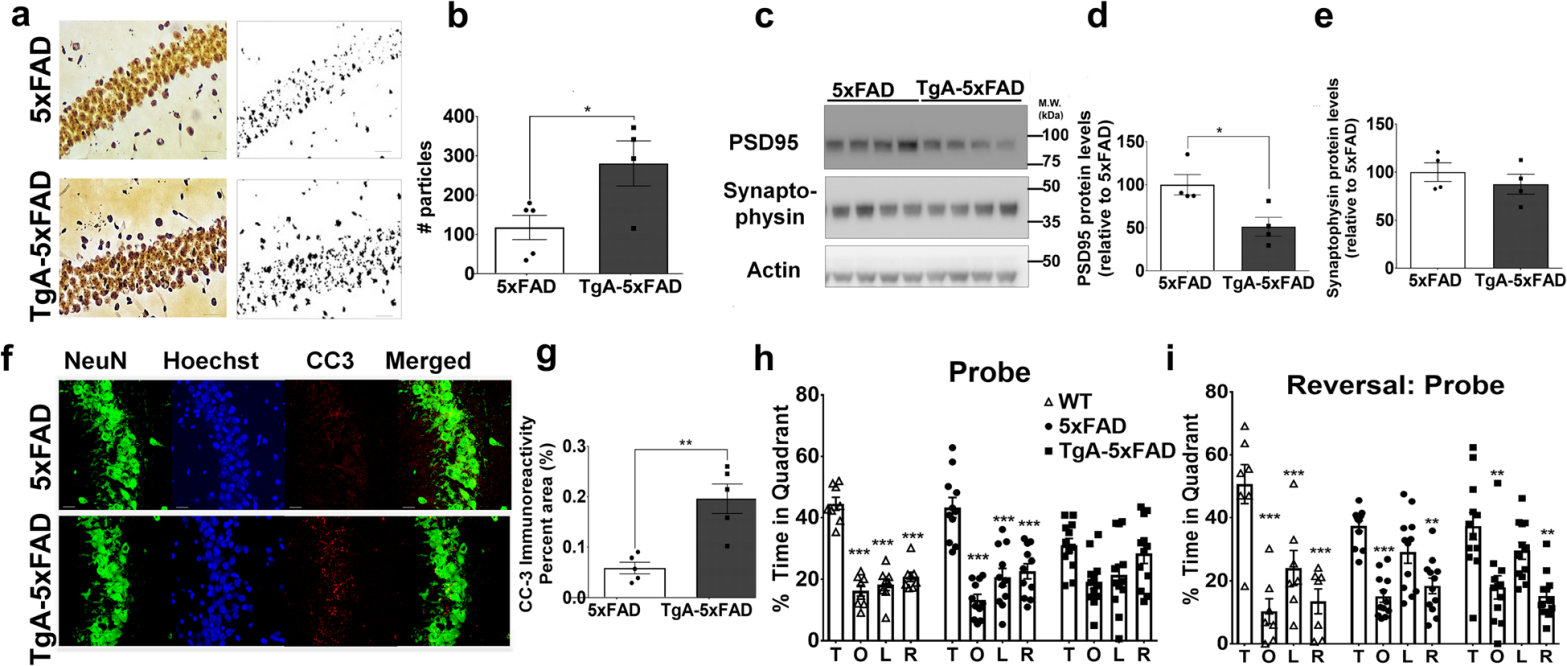
Increased neurodegeneration and memory deficits in 5xFAD mice overexpressing STI1. **a** Silver staining representative images (20X magnification) in the CA1 of the hippocampus of 3–5-month old 5xFAD and TgA-5xFAD male mice. Scale bar = 50 μm. **b** Quantification of number of silver particles (analyzed from thresholded image) across the whole hippocampus in 5xFAD and TgA-5xFAD mice. Three-four sagittal sections from at least four 5xFAD or TgA-5xFAD mice were used for all staining and immunostaining experiments. Black circles depict individual 5xFAD mice and black squares represent individual TgA-5xFAD mice. **c** Relative protein expression of synaptic markers PSD95 and synaptophysin in cortical lysates from 3 to 5-month old 5xFAD and TgA-5xFAD male mice and **d, e** densitometric quantification, normalized to actin loading control. **f** Cleaved caspase-3 (CC-3) representative images in the CA1 of the hippocampus (scale bars, 25 μm) and **g**, quantification of CC-3 immunofluorescence, presented as average percent area across the hippocampus. Data are presented as mean ± SEM, and data presented in **b**, **d**, **g**, **e** were analyzed using unpaired t-test. *p < 0.05, ** p < 0.01. **h** Mice were trained for four days on the spatial Morris Water Maze. On the fifth day (Probe trial), the target platform was removed and memory for what quadrant of the pool the target platform was placed, was quantified as the percentage of time in each quadrant. This was assessed in WT (TgA^−^-5xFAD^−^, *n* = 7), 5xFAD (*n* = 12) and TgA-5xFAD (n = 12) mice. **i** Two days following the Probe trial, animals were put on the “reversal” phase of the task, in which the target platform was placed in a different quadrant. Animals were trained for four days then memory was probed again and is denoted as the Reversal: Probe. T = Target, O = quadrant Opposite to target quadrant, L = quadrant to the Left of the target quadrant, R = quadrant to the Right of the target quadrant. All MWM data were analyzed using Two-Way Repeated Measures ANOVA with appropriate Tukey’s post hoc multiple comparisons. The * symbols in **h, i** represent Tukey’s post hoc multiple comparisons analysis, comparing percent time spent in the target quadrant (T) comparing the O, L, or R quadrants, within each genotype. *p < 0.05, ** p < 0.01, *** p < 0.0001 for within genotype comparisons for the MWM task. Raw data available at: 10.6084/m9.figshare.12115716

Aggregation of Aβ in plaques has been proposed to sequester more toxic and soluble Aβ species and serve as a reservoir of inert proteins [61, 62]. Given that in primary hippocampal neurons STI1 is neuroprotective and reduces AβO-mediated toxicity (Fig. 1 and [29]) and *C. elegans* experiments showed that STI1 prevented Aβ toxicity in body wall muscle, we performed a number of assays to assess whether 3–5-month-old TgA-5xFAD mice present increased resilience to Aβ-toxicity. Firstly, we used the FD NeuroSilver staining kit, which labels degenerating neurons (axons and soma) [50, 63–66]. In the hippocampi of male mice, we found significantly more silver particles in TgA-5xFAD mice, compared to their 5xFAD controls (Fig. 4a, b, *p* = 0.033), suggesting that STI1 did not confer protection. Synapse loss is also a consequence of Aβ oligomerization and aggregation, thus we evaluated the levels of synaptic proteins, such as the post-synaptic density marker PSD95, and the pre-synaptic marker synaptophysin [60, 67]. PSD95 was reduced by 50% in TgA-5xFAD hippocampal tissue (Fig. 4c, d, *p* = 0.023), however no significant changes were detected between genotypes for synaptophysin at this age (Fig. 4c, e, *p* > 0.1). Cleaved caspase-3 immunoreactivity was also two-fold higher in the whole hippocampus of TgA-5xFAD mice when compared to littermate 5xFAD mice (Fig. 4f, g, *p* = 0.002). Taken together, elevation of STI1 (combined with Hsp90) was not neuroprotective, but rather these results suggest increased accumulation of Aβ and plaque deposition heightened downstream toxicity.

Given the findings that TgA-5xFAD mice have higher levels of amyloid burden and neurotoxicity compared to littermate 5xFAD mice, we next sought to determine if there were any behavioural consequences. We addressed this by assessing mouse learning and memory in the hippocampus-dependent spatial MWM task. We used 6-month-old mice and found no significant differences between genotypes (wild type (WT) of mixed B6SJL/C57BL6/J background vs 5xFAD vs TgA-5xFAD) on time to find platform (*p* > 0.1), distance travelled to platform (p > 0.1), mean speed travelled (p > 0.1), or efficiency of path traveled to platform (p > 0.1) during the learning phase of the MWM. During the probe trial, which assesses spatial reference memory, WT and 5xFAD mice spend more time in the target quadrant (Fig. 4h, *p* < 0.001 for both WT and 5xFAD comparisons of T vs O vs L vs R) and there was no significant difference between time spent in target quadrant between 5xFAD and WT mice (Fig. 4h, *p* > 0.1), suggesting that at this age the mixed background 5xFAD mice used here did not yet present deficits in reference memory. A similar result was previously reported by Schneider et al. [68] when examining 6-month-old 5xFAD male mice. In contrast, TgA-5xFAD mice did not show preference for the target quadrant (T vs O *p* = 0.076, whereas T vs L or T vs R p > 0.1), and these mice spent significantly less time than WT and (*p* = 0.011) and 5xFAD mice (*p* = 0.043) in the target quadrant. Hence, TgA-5xFAD mice have worse spatial memory than 5xFAD mice. We then performed the reversal component of the task, which assesses behavioural flexibility. This entails changing the target quadrant where the escaping platform is located, training the mice for an additional 4 days, and then re-probing the memory on the fifth day for the new target quadrant. In this reversal task, when compared to WT control mice, both 5xFAD mice and TgA-5xFAD mice spent less time in the target quadrant than controls (Fig. 4i, *p* = 0.03 and *p* = 0.029, respectively), with no difference between 5xFAD and TgA-5xFAD mice (*p* > 0.1). These results demonstrate that both 5xFAD and TgA-5xFAD mice have deficits in behavioural flexibility in comparison to WT control mice. Interestingly, when preference for the target quadrant was evaluated, both 5xFAD and TgA-5xFAD mice showed increased preference for target quadrant compared to the opposite and right quadrant, suggesting that increased training potentially helped these mutants to solve the task.

### Unaltered APP processing but differential expression of enzymes involved in Aβ-degradation

**Fig. 5:**
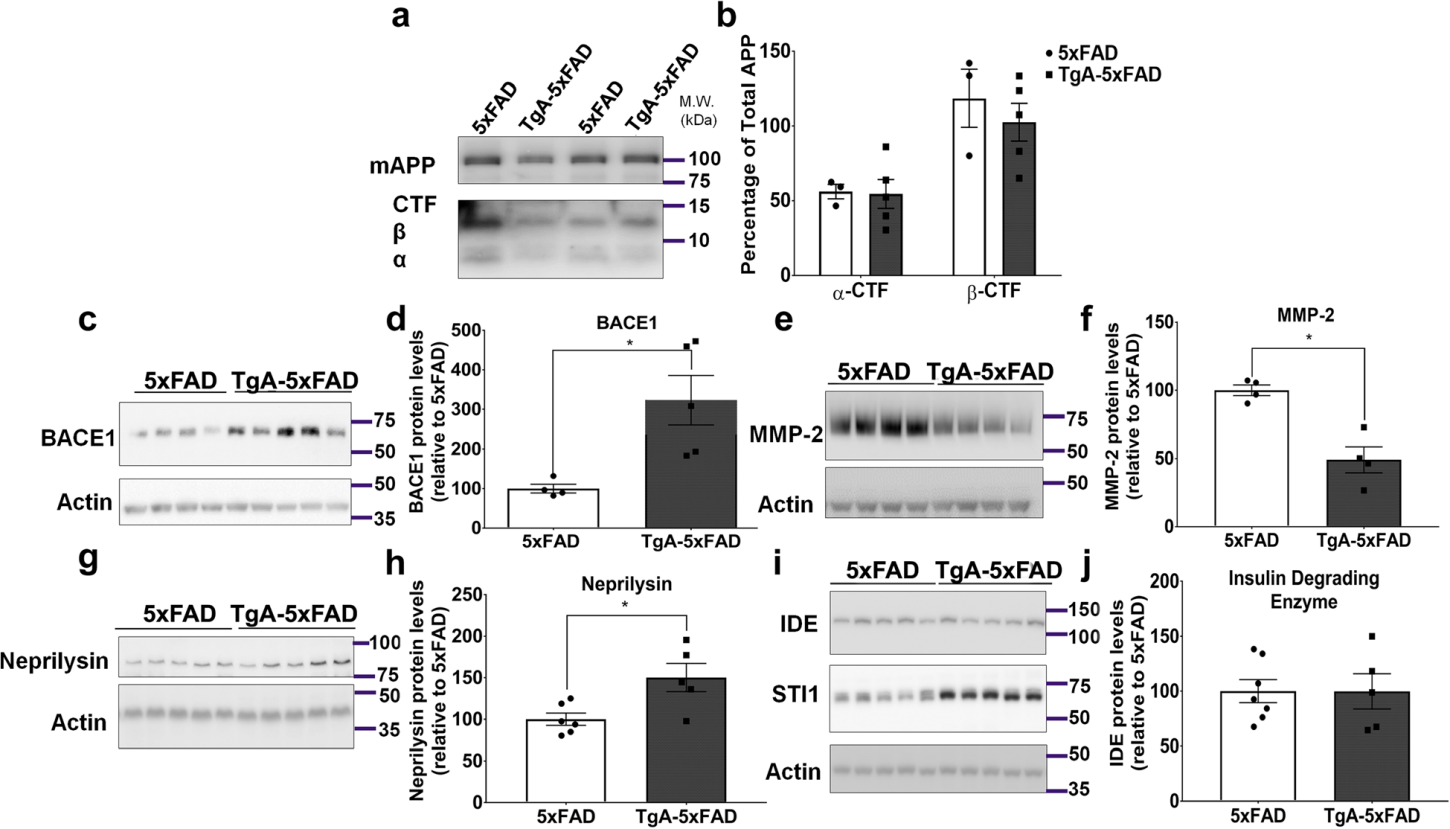
Overexpression of STI1 in 5xFAD mice does not affect APP processing, however levels of Aβ regulating enzymes are altered. **a** Representative Western blot for membrane bound full length APP and C-terminal APP fragments in cortices of 3–5-month old male 5xFAD and TgA-5xFAD mice. **b** Densitometric quantification of C-terminal fragment protein levels relative to full length APP, presented as percentage of total full-length APP [Signal intensity of the α or β fragment/signal intensity of full-length APP] × 100). At least 3 mice/genotype were used for experiments. Data were analyzed with Two-way ANOVA with Sidak’s post-hoc comparisons. On each graph, black circles represent individual 5xFAD mice and black squares are individual TgA-5xFAD mice. **c** Protein levels of BACE1 in cortical tissue from 3 to 5-month old 5xFAD and TgA-5xFAD mice and **d**, densitometric analysis. **e** Representative immunoblot and quantification **f**, for Aβ-degrading enzyme, matrix-metalloproteinase-2 (MMP-2). **g** Representative immunoblot and **h** quantification, for Aβ-degrading enzyme neprilysin. **i** Representative immunoblot and **j** quantification, for Aβ-degrading enzyme, Insulin-degrading enzyme (IDE). For C-J, N = 5 mice/genotype. Data are presented as mean ± SEM. Experiments were analyzed using unpaired t-test. *p < 0.05. Raw data available at: 10.6084/m9.figshare.12115758

The elevated levels of insoluble Aβ and acceleration of plaque pathology due to increased STI1 levels may be caused by a number of factors. These include increased APP levels, changes in APP metabolites, changes in the enzymes or processes involved in Aβ metabolism, decreased degradation of Aβ, or a combination of these factors. Given the diverse roles of intracellular and extracellular STI1 and Hsp90, it is conceivable that more than one mechanism is required to increase insoluble Aβ. We evaluated the levels of full-length APP in the membrane bound fraction and found no difference between genotypes for full-length APP, or α and β C-terminal fragments (Fig. 5a, b, *p* > 0.1), suggesting that the increased amyloid deposition observed in Fig. 3 is not due to increased APP levels or processing. In contrast, the levels of enzymes known to regulate Aβ cleavage or degradation were affected by STI1 overexpression. The β-secretase BACE1, the pro-amyloidogenic enzyme that cleaves APP to form the β-CTF [69–71] was upregulated three-fold in TgA-5xFAD mice compared to 5xFAD mice (Fig. 5c, d, *p* = 0.016). We examined the levels of BACE1 in WT vs TgA mice (C57BL/6 background) and found that BACE1 was also significantly elevated in TgA cortical lysates, however only by 1.5-fold (data not shown). MMP-2, a matrix metalloproteinase known to interact with both Hsp90 and STI1 [72, 73] can degrade Aβ, and overexpression of this protein lessened amyloid burden in 5xFAD mice [74]. We found a 50% reduction in MMP-2 protein levels (antibody for pro and activated forms of MMP-2) in TgA-5xFAD mice compared to 5xFAD controls (Fig. 5e, f, *p* = 0.0026). Neprilysin, a well-studied Aβ-degrading enzyme, that is reduced in the hippocampus and cortex of AD mice and humans [75, 76], was slightly increased in TgA-5xFAD mice by about 1.5-fold, compared to 5xFAD controls (Fig. 5g, h, *p* = 0.018). Lastly, we measured protein levels of insulin-degrading enzyme – which is proposed to have chaperone-like activity when degrading Aβ-peptides [77, 78], and found no difference between TgA-5xFAD and 5xFAD mice (Fig. 5i, j, *p* > 0.1). These results suggest multiple changes in levels of proteins that participate in amyloid generation and degradation could lead to increased production or decreased clearance of Aβ due to increased STI1 (with the compensatory increase in Hsp90β as well).

**Fig. 6:**
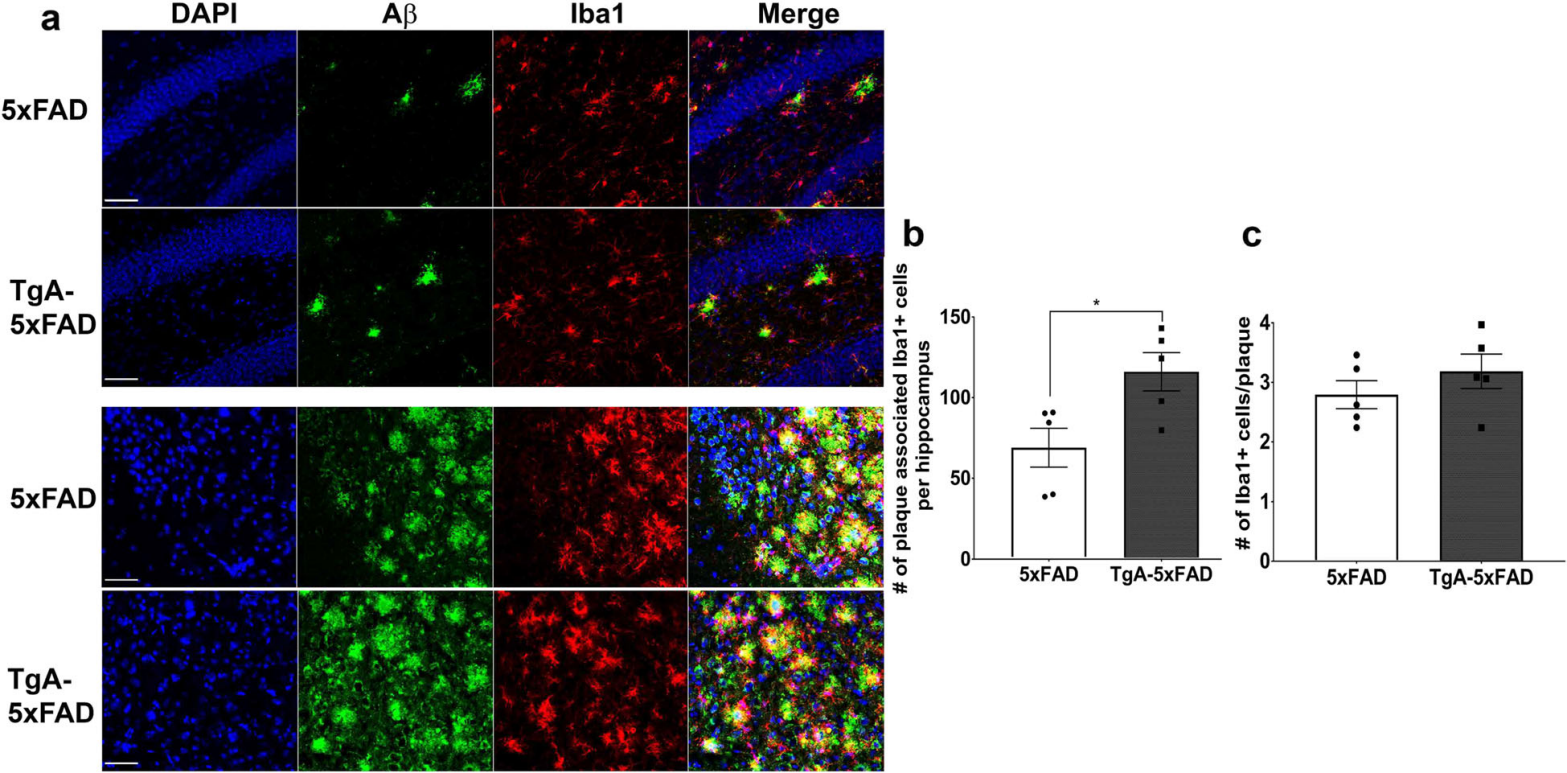
Microgliosis in the hippocampi of TgA-5xFAD mice. **a** Representative confocal micrographs from the dentate gyrus (DG) and subiculum immunolabelled for Aβ (anti-6E10) and microglial marker Iba1. Analyses **b & c** are from 5 individual mice with four sections being imaged per mouse at 40X magnification using confocal microscopy. Two images along the DG, one along the CA3, one along the CA1 and one in the subiculum were analyzed for number of plaques, number of microglia and number of microglia around plaques. **b** Quantification of the sum (average across sections and mice) number of microglia in contact with plaques across the whole hippocampus. **c** Quantification of the average number of microglia in contact with each plaque across all subfields. Scale bars = 50 μm. Data are presented as mean ± SEM. Experiments were analyzed using unpaired t-test. *p < 0.05. Raw data available at: 10.6084/m9.figshare.12115782

Another mechanism by which amyloid peptides are degraded, is clearance by microglia [79, 80]. We addressed whether 3–5-month-old TgA-5xFAD mice could present altered levels of microglia in contact with plaques, since microglia are known to phagocytose Aβ [79, 81, 82]. Immunostaining of brain tissue for microglia (Iba1) [83] and Aβ ([6E10, (Fig. 6a)] revealed that a significantly higher number of microglia were closely associated with plaques across the whole hippocampus in TgA-5xFAD vs 5xFAD mice (Fig. 6b, *p* = 0.023), likely due to the presence of more plaques in TgA-5xFAD mice (representative images of dentate gyrus and subiculum shown in Fig. 6a). However, the average number of microglia in contact with an individual plaque did not differ between 5xFAD and TgA-5xFAD mice (Fig. 6c, p > 0.1). Relative to neurons, microglia express very low levels of STI1 and Hsp90, as revealed by single-cell RNAseq [84], thus our results suggest that increased levels of STI1 did not impact microgliosis around plaques and that the increased deposition of Aβ is not due to decreased microglia in contact with plaques.

### Accumulation of extracellular STI1 and Hsp90 around Aβ dense core, senile plaques

**Fig. 7:**
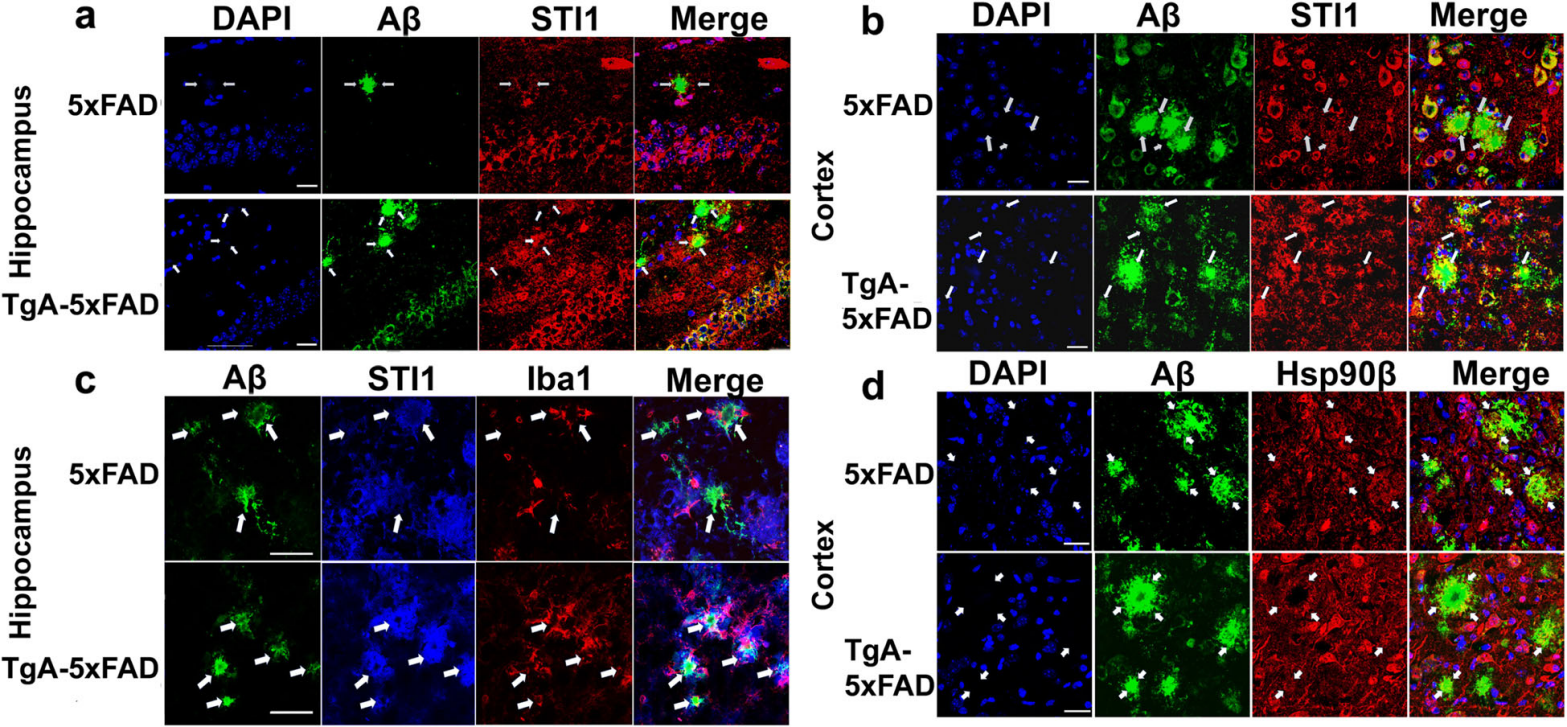
Extracellular STI1 deposits are found in dense core and neuritic plaques in 5xFAD mice. **a** Representative confocal images (63X magnification) of Aβ (anti-6E10, green), STI1 (red) and nuclei labelled with DAPI (blue) in the CA1 region of the hippocampus of 3–5-month old male 5xFAD and TgA-5xFAD mice. Images are Z-stack projections composed of images including Aβ plaques. White arrows indicate areas where extracellular STI1 is visualized within the Aβ deposits around the core of the plaque. Scale bars = 25 μm. **b** Aβ plaques and STI1 immunolabelling in the cortex of 3–5-month old 5xFAD and TgA-5xFAD male mice. Scale bars = 25 μm. **c** Aβ (6E10, green), STI1 (blue) and Iba1 microglial marker (red) labelling in the subiculum of 3–5-month old male 5xFAD and TgA-5xFAD mice. White arrows indicate areas where extracellular STI1 is visualized within the Aβ deposits around the core of the plaque. **d** Representative images of tissue labelled with DAPI (blue) Aβ (6E10, green), Hsp90β (red) in the cortex of 3–5-month old male 5xFAD and TgA-5xFAD mice. White arrowheads indicate areas where extracellular Hsp90β deposits are colocalizing within plaques. Three-four sagittal sections from at least three 5xFAD or TgA-5xFAD mice were used for all immunostaining experiments. Scale bars = 25 μm

The above experiments suggest that STI1 potentially favours increased production and decreased degradation of Aβ, given the increase in BACE1 and decreased levels of enzymes that can degrade amyloid. STI1, Hsp70 and Hsp90 are secreted by a variety of cells and they are found in the extracellular milieu [25, 85–87], plasma [88] and in principle they could directly contribute to the regulation of Aβ aggregation. Notably, glia secreted Hsp70 reduces neuronal stress in LA-N-5 neuroblastoma cells [89], reduces Aβ toxicity in *Drosophila* [90], and extracellular Hsp70 lessens Aβ-oligomerization and attenuates Aβ toxicity in N2A cells [91]. Moreover, Hsp90 has been previously shown to decrease Aβ fibrillization in vitro [56, 92]. These results may suggest that STI1 could be closely associated with plaques, which we tested by using immunofluorescence confocal microscopy. We performed unbiased, random imaging of STI1 and Aβ plaques in the hippocampus and cortex of 3–5-month-old 5xFAD and TgA-5xFAD mice. We observed STI1 around the dense core of Aβ plaques (anti-6E10 antibody), representing a ring-like structure in both 5xFAD and TgA-5xFAD mice (Fig. 7a). STI1 around plaques did not seem to be located in microglia (Fig. 7c), as there is little colocalization between STI1 staining and Iba1+ glial cells, or nuclear marker DAPI (Fig. 7a-c). STI1 co-localization with the Aβ immunoreactive neuritic plaques/cored plaques can be observed in both 5xFAD and TgA-5xFAD mice in both the cortex and hippocampus (Fig. 7a, b), but it is more prominent in TgA-5xFAD. Likewise, we observed co-localization of Hsp90β with dense-core plaques, in both 5xFAD and, increasingly more discernable, in TgA-5xFAD mice (Fig. 7d). These results suggest that extracellular STI1 and Hsp90β can accumulate in Aβ plaques.

**Fig. 8:**
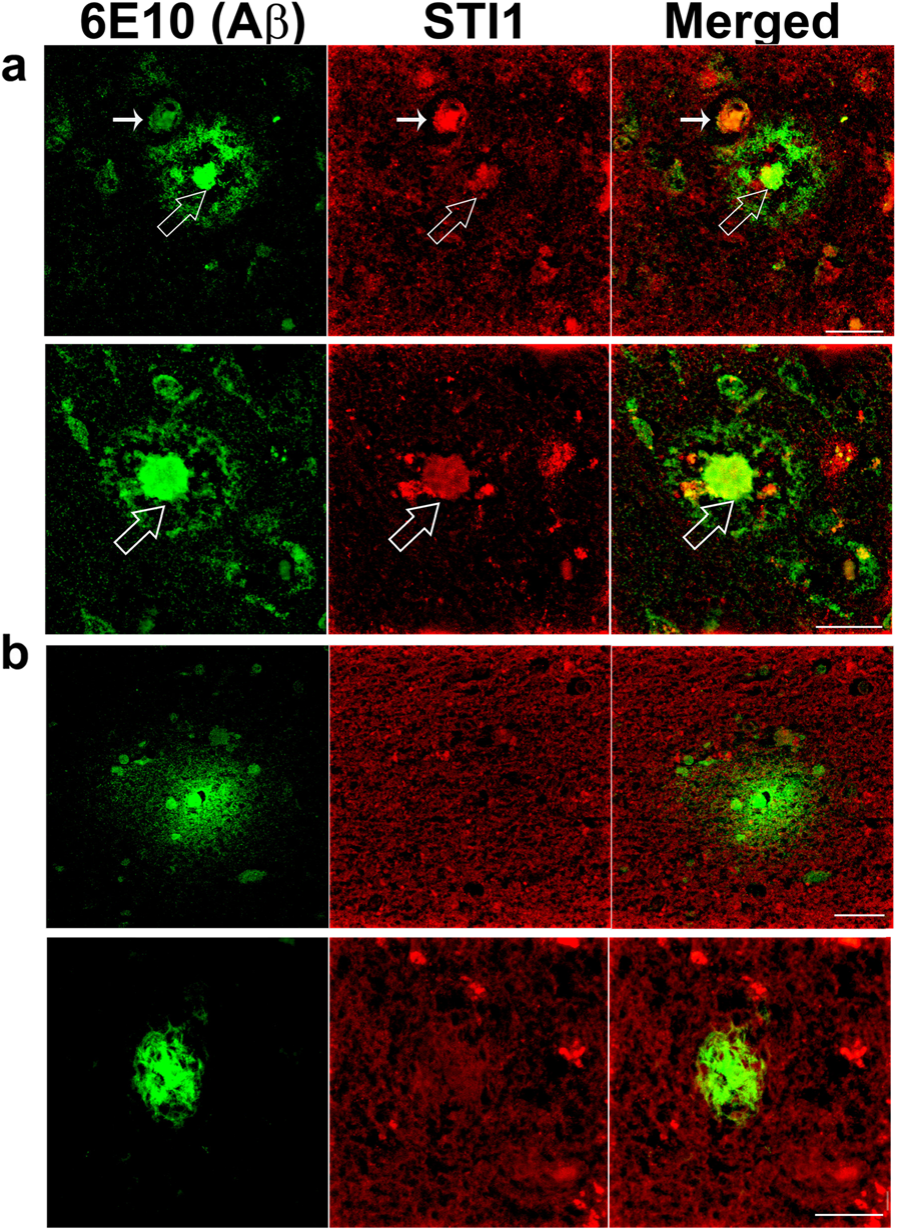
Dense-core plaques in human Alzheimer’s patients have extracellular STI1 deposits. **a** Representative images of mature dense core Aβ plaques (63X) from five AD patients in temporal and parietal cortex. **b** Representative images of diffuse Aβ plaques images from five AD patients (63X). Green is for Aβ (anti-6E10), red for STI1. Scale bars = 25 μm. Large white arrowhead show STI1 and Aβ colocalization, small filled white arrowheads show STI1 + Aβ immunoreactive blood vessels

Importantly, we verified that STI1 is also present in plaques in human AD brains. Immunofluorescence imaging of STI1 and Aβ in five AD patient brains showed significant STI1 labeling in what seemed to be more mature plaques (a minimum of 5 found in each section) with dense cores of Aβ (Fig. 8a; a minimum of 3 distinct sections labelled and plaques imaged). Conversely, we also observed several examples of less mature plaques (at least 10–20 per brain section) in which STI1 was not accumulated (Fig. 8b). Blood vessels that were positive for Aβ also showed STI1 immunoreactivity (small white filled arrow in Fig. 8a).

## Discussion

The present experiments revealed the relationship between STI1 and Hsp90 in worm and mouse AD models of amyloidosis in vivo. We confirmed and extended previous results demonstrating that in cultured neurons, overexpression of STI1 prevented Aβ toxicity via extracellular mechanisms. We also report an inverse relationship between levels of Hsp90, STI1 and Aβ toxicity in worms, suggesting that increasing the levels of Hsp90 and STI1 is protective.

In contrast, in mammals, endogenous elevation of STI1 and compensatory ectopic increase in Hsp90β resulted in much more complex effects in vivo. In general, we show that increasing STI1 and Hsp90 levels in mice overexpressing human APP with mutations that favour the generation of toxic Aβ peptides (5xFAD), escalated the accumulation of insoluble protein aggregates. Importantly, measures of cellular toxicity in brain tissue, and mouse behavioral tests suggest that increased insoluble Aβ plaque burden are linked to worse outcome in TgA-5xFAD mice. However, given the resulting increase of Hsp90 due to overexpression of STI1, it remains unknown if overexpression of Hsp90β alone in 5xFAD mice would lead to the same increase in amyloid burden or impact levels of STI1 in vivo. In vitro neuronal assays with recombinant Hsp90 and STI1 by Maciejewski et al. [48] revealed that surplus addition of Hsp90 in the presence of increasing concentrations of STI1 prevented STI1 neuroprotection against AβOs. These results suggest that excessive levels of Hsp90 interferes with STI1 binding to PrP^C^, which could explain why more neurodegeneration was found in TgA-5xFAD mice.

Different types of Aβ aggregates may act in different ways to affect neurons. Numerous recent experiments have focused on the toxicity of Aβ oligomers and demonstrated that these toxic species interact with the prion protein to trigger maladaptive neuronal signalling [19, 29, 93–95]. In vitro data suggest that extracellular STI1 is beneficial for neurons either by leading to increased protein translation [96], neuritogenesis [26], or protecting neurons against a number of chemical or environmental insults [25, 26, 28]. Indeed, we found that secreted STI1 by TgA neurons was responsible for the enhanced resilience against AβOs, since blocking STI1 with an anti-STI1 antibody abolished this protection.

In addition to the toxic effects of AβOs, the accumulation of insoluble forms of Aβ can trigger ER stress [54, 97–99] and burden cellular protein quality control. Interestingly, transcriptomic analyses of AD patients’ brains identified STI1 as a regulator of the unfolded protein response [31]. In yeast, which have highly conserved STI1 and Hsp90 genes, STI1 activity seems to be vulnerable to excessive amounts of accumulated exogenous proteins [42], which then disturbs chaperone network activity. Moreover, increased levels of STI1 decreased amyloid toxicity in yeast, by regulating their spatial distribution and toxic assembly [35]. These results suggest that increasing the levels of STI1 and Hsp90 seem to help certain organisms, such as yeast and worms, whose Aβ is not secreted and remains intracellularly [35, 100] to deal with the increased proteotoxicity of amyloid proteins. Although our results in *C. elegans*, an organism that does not have a homologue for the prion protein, are in line with the in vitro experiments [29, 101], it is clear that in mice the extracellular effects of STI1 via the prion protein do not seem to be as relevant as other actions, such as STI1 modulation of proteostasis and protein aggregation.

STI1 and Hsp90 are secreted by a myriad of cells and have the potential to be found extracellularly in the mammalian brain [25, 85–87, 102]. Regulation of extracellular Hsp90 by co-chaperones, and extracellular Hsp90 chaperone activity is not well studied, but it was recently found that extracellular Hsp90 is regulated by co-chaperones Aha1 and TIMP2 to regulate their client protein MMP-2 [103]. These results raise the possibility that extracellular STI1 could indeed be regulating and altering the Hsp70/Hsp90 machinery. Moreover, the increased levels of STI1 and consequent increase in Hsp90 appear to contribute to augmented Aβ burden in mice in different ways. The levels of BACE1 were increased three-fold, whereas MMP-2, an important enzyme for amyloid β degradation, and an Hsp90 client, was significantly decreased. However, APP levels were not different between 5xFAD and TgA-5xFAD mice. We detected no evidence that microglia close to plaques were altered, suggesting that increased STI1 levels may not affect this mechanism of plaque removal.

There has been debate in the literature as to whether amyloid plaques are indeed toxic or may be a means to protect cells against Aβ-peptide and AβO toxicity [61, 62, 104, 105]. Our results show that the amplified amyloid burden in TgA-5xFAD correlates with increased neurodegeneration, post-synaptic density loss, increased apoptosis, as well as worse performance of mice in the spatial MWM. Hence, it seems that at least for the 5xFAD mouse line, the increase in plaques was not protective and Aβ oligomer toxicity does not play a major role in the toxicity, given that this should be blocked by extracellular STI1.

The role of Hsp70/Hsp90 and STI1 in other types of protein misfolding diseases is starting to be investigated and warrants further analysis. Tau is a client of Hsp90 and inhibition of Hsp90 to stimulate Hsp90 re-folding pathway seems to favour degradation of overexpressed misfolded tau and phospho-tau [106]. Hsp90 co-chaperones STI1, Hsp40, CHIP, p23, and Pin1 were all necessary for mediating tau re-folding and/or degradation. Furthermore, STI1/Hop loss of function in *Drosophila* overexpressing the full-length 2 N/4R isoform of wild-type human tau in the eye, resulted in significant loss of retinal cells [107]. Recently, Inda et al. [40] demonstrated that in AD, chaperones associate to form large stable complexes, termed epichaperomes, that no longer regulate client protein function. These epichaperomes impaired protein connectivity, proteostasis, and synaptic plasticity in their AD mouse models [40], which could be corrected by disassembly of epichaperomes by an Hsp90 inhibitor. Since Hsp90, Hsp70 and STI1 are all members of these epichaperomes in AD tissues, it may be important to test in the future how increased or decreased levels of STI1 regulate epichaperomes.

Hsp90, along with other small Hsps have been previously found to co-localize with Aβ plaques [80, 108–111], however it is not fully elucidated whether this is a result from cellular debris or if the plaques sequester chaperones. We found STI1 and Hsp90 immunolabelling in dense core and neuritic plaques – the type of plaques that are associated with late stage AD and are typically surrounded by glial cells [112]. Interestingly, we similarly found that in AD patients, STI1 accumulates only within the dense core of senile plaques. Deposits of chaperones in protein aggregates may disturb chaperone function, suggesting that molecular chaperone activity may be impaired in AD, potentially affecting a number of client proteins.

In summary we found a striking difference between the actions of Hsp90 and STI1 in *C. elegans* and in mice. Overall, the observations presented here suggest a complex relationship between Hsp90, STI1, and Aβ peptides in mammals. These results support the notion that increased STI1 levels found in human AD patient brain tissue [29], may contribute to amyloidogenesis and paradoxically increase toxicity in vivo.

## Data Availability

Datasets were deposited in Figshare, and are publicly available. Figure 1: 10.6084/m9.figshare.12115614 Figure 2: 10.6084/m9.figshare.12115704 Figure 3: 10.6084/m9.figshare.12115713 Figure 4: 10.6084/m9.figshare.12115716 Figure 5: 10.6084/m9.figshare.12115758 Figure 6: 10.6084/m9.figshare.12115758.
